# Medical care for transgender individuals at a hospital in southern Brazil: why do they drop out from our service?

**DOI:** 10.3389/fpubh.2024.1254875

**Published:** 2024-07-16

**Authors:** Fernanda Guadagnin, Karine Schwarz, Dhiordan Cardoso da Silva, Leonardo Romeira Salati, Vinicius Kayser, Maria Inês Rodrigues Lobato

**Affiliations:** ^1^Department of Psychiatry, Gender Identity Program at Hospital de Clínicas de Porto Alegre, Porto Alegre, Brazil; ^2^Graduate Program in Psychiatry and Behavioral Sciences, Federal University of Rio Grande do Sul, Porto Alegre, Brazil; ^3^Postgraduate Program in Medical Sciences: Endocrinology, Federal University of Rio Grande do Sul, Porto Alegre, Brazil; ^4^Federal University of Health Sciences of Porto Alegre, Porto Alegre, Brazil

**Keywords:** transgender persons, gender identity, health services accessibility, health services for transgender persons, patient dropout

## Abstract

**Objective:**

The objective of the study was to investigate dropout rates and discern potential factors contributing to the discontinuation of treatment provided to transgender individuals by the Transdisciplinary Gender Identity Program at the Hospital de Clínicas de Porto Alegre (PROTIG).

**Methodology:**

This study employs a descriptive, cross-sectional, retrospective design to analyze socio-demographic and clinical data obtained from medical records of patients treated at PROTIG between 2000 and 2018. A structured form, devised by PROTIG’s professional team, was utilized to extract and evaluate several variables including: age, gender, education level, diagnosis of F64 according to the International Classification of Diseases (ICD-10: Version: 2010), clinical comorbidities (coded by ICD-10), laboratory diagnosis of sexually transmitted infections, distance between patients’ residence and the hospital, and year of entry into PROTIG. The patient cohort was stratified into two categories based on their duration of attendance: dropout (defined as attendance for up to 365 days) and non-dropout (attendance exceeding 365 days). Categorical variables between dropout and non-dropout groups were compared using Pearson’s chi-square test. Additionally, Poisson regression analysis was utilized, employing a 95% confidence interval (CI) and setting the significance level at 0.05.

**Results:**

The study included a total of 888 patients accessing PROTIG, with 275 (31%) classified in the dropout group. Of the patient population, 65.5% (*n* = 582) self-identified as transgender women, while 34.5% (*n* = 306) identified as transgender men. Significant differences were noted between the dropout and non-dropout groups. Specifically, differences were noted among transgender women (*p* < 0.001), individuals with lower levels of education (*p* < 0.001), those with fewer diagnoses classified under ICD-10 as F64 (*p* < 0.001), individuals exhibiting fewer clinical comorbidities recorded in ICD-10 (*p* < 0.001), and those who commenced inclusion in PROTIG after 2010 (*p* < 0.001).

**Conclusion:**

There exists a notable rate of treatment discontinuation among individuals receiving care at PROTIG, with statistically significant variances observed between groups. We posit potential rationales for this discontinuation, informed by care experiences and feedback from group attendees: Increased accessibility to outpatient services in our jurisdiction for Transgender Care, along with heightened societal awareness of gender identity fostering diverse gender expression avenues devoid of reliance on gender-affirming surgical interventions.

## Introduction

Medical assistance for transgender individuals in Brazil was initiated in 1998 following an order from the Brazilian Medical Council, and it was officially incorporated into the Brazilian public health system in 2013 (1, 2). Despite notable advancements in the availability of Transgender Care services over the past decade, our institution located in the state of Rio Grande do Sul remains the exclusive facility providing gender-affirming surgery in southern Brazil. The Hospital de Clínicas de Porto Alegre (HCPA), through its Transdisciplinary Gender Identity Program (PROTIG), has delivered comprehensive services tailored to transgender individuals, encompassing gender transition via surgical interventions. To date, the program has conducted evaluations on more than 1,000 patients, with an average of two surgeries performed monthly, resulting in over 300 surgical procedures (3, 4).

The healthcare team dedicated to transgender individuals at HCPA consists of two urologists, one gynecologist, one plastic surgeon, two psychiatrists, one psychologist, one endocrinologist, one nurse, and one social worker, in adherence to directives set forth by the Brazilian Ministry of Health. Within the framework of PROTIG, following a two-year period of medical support comprising individual and group consultations, patients are subsequently referred to surgical teams for comprehensive examinations and preoperative assessments.

The obstacles to accessing our program are widely acknowledged in Brazil. This stems from the structure of the public health system, which mandates referrals following medical evaluations at health centers. As previously mentioned, being the sole provider of surgical transition procedures in southern Brazil, spanning three states with a combined population of approximately 30 million people, has resulted in a substantial waiting list (5). Consequently, the unexpected rate of patient attrition from our medical assistance has been a source of surprise for our team.

Treatment discontinuation is a prevalent issue across various medical programs globally, including in Brazil, particularly in the context of long-term treatments such as those for tuberculosis, leprosy, or HIV. Factors such as socioeconomic status, education level, barriers to healthcare access, demographic characteristics, as well as the adverse effects of medications and substance abuse, may all play significant roles in contributing to treatment dropout (6–8). Long-term assistance programs, such as those for obesity, borderline personality disorder, and drug abuse, are also associated with high dropout rates. In these instances, psychological factors may play a role in premature treatment discontinuation (9–11). Another study, aimed at investigating dropout rates and associated factors among patients with drug dependence, revealed that, from the patients’ viewpoint, motivational incongruities were deemed the primary cause for either refraining from initiating or discontinuing treatment (12).

Based on the literature we have reviewed, no published studies were found concerning dropout rates from transgender affirmative surgery treatment. Hence, this study aims to explore potential reasons for dropout from medical assistance provided to transgender individuals at PROTIG. This will be achieved by analyzing socio-demographic and clinical data extracted from medical records spanning from 2000 to 2018.

## Methods

A method of retrospective, cross-sectional analysis, of medical records was developed by the multi-professional team of PROTIG that included factors to be search such as demographic data (including age, gender, educational level, home address), sexually transmitted infections (STI) exams, F64 (ICD-10), clinical comorbidities ICD-10 (including any clinical and psychiatric diagnoses) and year of entry into PROTIG (13). Using these variables, we investigated possible correlations with dropout rates in PROTIG. The sample studied was followed in our hospital from 2000 to 2018. Incomplete medical record data was used as an exclusion criterion. Upon enrollment in PROTIG, individuals provide written informed consent for the utilization of their data in studies and research, ensuring patient confidentiality. As part of the research protocol, a formal request was submitted to HCPA, specifying the study variables, to electronically extract data from medical records. For statistical analysis, the sample was stratified into two cohorts: the Dropout group, consisting of individuals who ceased treatment within 365 days following the initial consultation, and the Non-dropout group, comprising those who continued treatment beyond this timeframe. This study received approval from the Research Ethics Committee of Hospital de Clínicas de Porto Alegre (HCPA), under protocol number 2019–0115.

### Statical analysis

Descriptive and statistical analyses were performed using the SPSS software program (version 18.0). The age value was calculated by mean and standard deviation. For the present study, eight variables were selected from the database. Categorical variables were expressed by frequencies and percentages: age, home address, gender, F.64 diagnosis, education level, clinical comorbidities, STIs (HIV, VDRL, HBsAG), and year of entry into PROTIG. The analysis involving the comparison of categorical variables between the two groups was performed using Pearson’s chi-square. Following this, a multiple Poisson regression with robust variance was performed including all variables that have *p* < 0.05 at bivariate analysis to estimate the prevalence rate ratio. The aim was to estimate the prevalence ratios and their corresponding 95% confidence intervals.

## Results

The total sample consists of 889 individuals. One of them was excluded because the medical record data was incomplete. The mean age of the 888 patients who accessed our service was 27.96 ± 9.08 years at their initial consultation. Within the sample, the mean age for the dropout group (*n* = 275) was 28.15 ± 9.67 years, while for the non-dropout group (*n* = 613), it was 27.85 ± 8.80 years. Among the participants, 65.5% (*n* = 582) identified as transgender women, while 34.5% (*n* = 306) identified as transgender men. Of these, 275 patients (31%) discontinued treatment, comprising 62 (22.5%) transgender men and 213 (77.5%) transgender women.

The dropout group exhibited significant differences compared to the non-dropout group, characterized by a higher proportion of transgender women (*p* < 0.001), lower educational attainment (*p* < 0.001), fewer diagnoses classified under ICD-10 as F64 (*p* < 0.001), fewer clinical comorbidities according to ICD-10 (*p* < 0.001), and enrollment in PROTIG after 2010 (*p* < 0.001).

## Discussion

To our knowledge, this study represents the inaugural investigation delineating the discontinuation of transgender patients from a dedicated sexual affirmative surgical transition program. The primary objective of this study was to elucidate the factors contributing to dropout from medical interventions. There is a discernible rate of treatment cessation among individuals under care at PROTIG, with statistically significant differences noted between groups. After an extensive search through medical records of the patients that attended and later dropped out from PROTIG, we highlighted two possible reasons for this: greater availability of outpatient services in the state of Rio Grande do Sul for Transgender Care and a change in the social acceptance for gender identity variations that enables people to experience different gender expressions without the need for gender-affirming surgical procedures (14). Additionally, we do not discount motivational challenges linked to the intricate and irreversible nature of surgery (12).

Since 2010, according to data given by Department of Rio Grande do Sul, 14 specialized outpatient services for transgender care have been opened in our state. It is important to highlight that, despite the recent greater availability of outpatient services in our state, access to PROTIG continues to be particularly difficult under the current healthcare system. On average, medical assistance occurs at least 24 months after first accessing PROTIG. This waiting time is because we currently have few public services in Brazil that offer treatment for gender-affirming surgery, and we continue to be the only one in the South of Brazil to offer this service, despite the aforementioned improvements in public health services (15). Therefore, it is logical to consider that this may influence the drop out from our service since the advent of new Transgender services and the consequent decentralization of medical assistance in our state.

When we evaluated the data surveyed, we found a significant difference between the groups regarding gender and gender expressions, educational level, clinical morbidity, year of entry, and F64 ICD-10 diagnosis. The research group looked at eight hypothesized factors that could have influenced the patient’s attendance in PROTIG.

### Age

Our initial hypothesis that younger people would tend to abandon PROTIG more was not confirmed. There is a great variability of people who seek the program for treatment and since 2014 there has been service for groups of children and adolescents. Dropout from psychological treatment is a significant issue that substantially limits its effectiveness. In this study (16), the highest dropout rates from psychological treatment were observed among younger, single patients with lower education levels and incomes.

### Distance between home and hospital (home address)

Our facility receives patients from various regions within Rio Grande do Sul, as well as from other states across Brazil. Surprisingly, in our investigation, the distance between patients’ residences and the hospital did not emerge as a significant determinant of patient dropout, contrary to our initial expectations. One plausible explanation for this observation is the existence of a financial support program in Brazil dedicated to specialized healthcare services, including organ transplants, cancer treatment, and gender-affirming procedures, which facilitates patient transportation to access such services, including those offered by PROTIG. However, considering that the majority of our patients originate from economically disadvantaged backgrounds, we speculate that the influence of this factor may be underestimated (17). It is conceivable that there exists an unmeasured subgroup of patients who encounter difficulties in attending appointments, even when the distance is less than 100 kilometers. Unfortunately, this subgroup remained unidentified within the scope of our study (refer to Table 1).

**Table 1:** Variables by non-dropout and dropout groups.

Characteristics	TM* + TW**	None drop *N* (%)	Drop *N* (%)	Total	Statistical test
Age	888	613 Mean: 27.8 ±8.80	275 Mean: 28.1 ±9.67	888	*p* = 0.650
Clinical comorbidities	888	197 (46%) 0 ICD	233 (54%) 0 ICD	430 220 238	*p* < 0.001*
		198 (90%) 1 ICD	22 (10%) 1 ICD		
		218 (92%) 2 or more ICD	20 (8%) 2 ICD		
F.64 diagnosis	F.64-	236 (48%)	254 (52%)	490	*p* < 0.001***
	F.64+	377 (95%)	21 (5%)	398	
Education	Elementary school or illiterate	276 (65.1%)	148 (34.9%)	424	*p* < 0.001^***^
	Other	333 (73.7%)	119 (26.3%)	452	
Year of assessment	2000 2004	134 (85.4%)	23 (14.6%)	157	*P* < 0.001^***^
	2005 2009	134 (71.7%)	53 (28.3%)	187	
	2010 2014	169 (64.8%)	92 (35.2%)	261	
	2015 2018	176 (62.2%)	107 (37.8%)	284	

TM^*^, transgender men; TW^**^, transgender women; *Independent-samples Mann–Whitney *U* Test; ^***^Pearson Chi-Square.

### Diversity of gender and gender expressions and (F64 ICD-10 diagnosis)

One noteworthy finding in our study, potentially associated with the diversity of gender expressions, was the significant difference observed between the group diagnosed with F.64 (Gender Identity Disorder - ICD-10) as documented by the psychiatrist in the medical records, and the group in which the diagnosis was not established at any point during patient care (3). The dropout group may consist of individuals who exhibit little or no interest in undergoing gender-affirming surgical procedures (refer to Table 2), either due to the absence of gender dysphoria or the presence of non-severe gender dysphoria.

**Table 2:** Rate of dropout and non-dropout by gender.

	Gender	None drop *N* (%)	Drop *N* (%)	Total	Statistical test
Total	TM^*^	244 (79.7%)	62 (20.3%)	306	*p* < 0.001^***^
	TW^**^	369 (63.4%)	213 (36%)	582	
	Total	613 (69%)	275 (31%)	888	

TM^*^, transgender men; TW^**^, transgender women; ^***^Pearson Chi-Square.

Another finding was a significant difference between genders: more transgender women dropped out of the program than transgender men. We do not have a specific answer. We speculated that the social factor, “passing,” may have influenced this result. Both transgender men and women often come to our service with an existing satisfactory physical and social gender transition. However, in transgender women, the surgical implication (performing genital surgery) is intimate and its fulfillment or not, may not have repercussions in the social expression of gender (passing). This is unlike transgender men in which the surgical procedure of mastectomy, removes from public exposure a physical marker of females, which when not carried out in this group, generates constraints and some loss in social insertion.

### Educational level

A lower educational level in our study sample was a possible reason for greater drop-out rates. In this case, it is likely that the patients’ limitations regarding the understanding of the phenomenon of transsexuality/homosexuality and their greater difficulty in expressing their needs, may have caused misappropriated referrals to the PROTIG. When these patients were adequately informed about the type of care provided by us, they dropped out of our service (Table 1).

### Clinical comorbidities

The non-dropout group significantly differs (*p* < 0.001) from the dropout group in the context of clinical illness diagnosis (Table 1). This is not a surprise, since we know that this population has a risk to develop different clinical health problems, but the search for medical assistance may be delayed and is generally associated with social health barriers. When we analyzed the cluster F-ICD-10, we found that the most frequent clinical diagnoses were depression, anxiety, and suicide risk. These psychiatric syndromes are commonly associated with confrontation relating to social stigma, prejudice and marginalization suffered by this population. We have already reported our findings about this issue elsewhere (18–22). Probably the non-dropout group found in PROTIG, a place that assists these comorbidities besides offering surgical transition assistance. When we analyzed other ICD clusters, we found cluster B (HIV, 66 cases, and tuberculosis, 14 cases) to be the most frequent. Other diseases were far less prevalent.

### STIs diagnosis

We did not find, contrary to our expectations, significant differences between the clinical comorbidity of the two groups when considering STIs (VDRL 17.2% and Anti-Hbs 1.2%), despite the high prevalence of HIV (15.2%) when compared to the general population (Table 1). It is important to state that this search corresponds to laboratory results and not actual diagnoses requiring treatment. We interpret this finding as indicating a risk factor linked to prejudice, discrimination, and the challenging life trajectories experienced by individuals with minority gender identities (23, 24).

### Year of admittance to the PROTIG

We believe that the greater visibility in our society about transgender issues may also explain the recent increased rates of dropout (Tables 1, 3). The number of people who did not return in the last 3 years when compared to the first 5 years (108 vs. 23) has quadrupled (Table 1). Notably, these social changes (greater visibility + greater assistance care centers) regarding gender issues have been accompanied by changes in Brazilian legislation aiming to protect the Transgender population. For instance, the use of social names in oral treatment in educational institutions, use of bathrooms and other spaces, wearing of gender-assigned uniforms, and the guarantee of recognition of gender identity according to the transgender’s wishes (24). Protective resolutions were created in work relationships and the public health system in 2009, guaranteeing the right of the SUS user to be identified and attended to in health units by the name of their preference (25). This scenario of social and cultural changes that have occurred in Brazil in recent years, that is, increased social tolerance of non-binary gender expressions, may explain the reduction in interest in surgical gender transition.

**Table 3:** Results of multiple regression to evaluate risk factors for non-dropout and dropout groups.

	Prevalence ratio	Confidence interval	Sig.
Years of 2015 to 2018	2.080	(1.39–3.11)	<0.001
Years of 2010 to 2014	1.709	(1.13–2.56)	0.010
Years of 2005 to 2009	1.773	(1.15–2.73)	0.010
Years of 2000 to 2004	1		
F.64 diagnoses	0.112	(0.07–0.17)	<0.001
Secondary education*	0.738	(0.61–0.89)	0.002

*Poisson regression.

In conclusion, despite limitations concerning the lack of clinical information in medical records, we observed two possible conjectures that influenced the dropout from our service: One is the greater availability observed of outpatient services in the state of Rio Grande do Sul for Transgender care and the other is the greater visibility of the issues concerning gender identity variants that enables people to experience different gender expressions without the need for gender-affirming surgeries. It is reasonable to consider that these findings cannot be used as a general reference for the whole transgender population in the world due to cultural and social issues that are specifically related to Brazil.

## Data availability statement

The raw data supporting the conclusions of this article will be made available by the authors, without undue reservation.

## Author contributions

FG: Conceptualization, Data curation, Methodology, Writing – original draft, Writing – review & editing. KS: Funding acquisition, Methodology, Resources, Writing – original draft, Writing – review & editing. DC: Conceptualization, Data curation, Methodology, Writing – review & editing. LS: Writing – original draft, Writing – review & editing. VK: Conceptualization, Data curation, Formal analysis, Methodology, Software, Writing – original draft, Writing – review & editing. ML: Data curation, Methodology, Writing – original draft, Writing – review & editing.

## Ethics statement

The studies involving humans were approved by the Research Ethics Committee of Hospital de Clínicas de Porto Alegre (HCPA), under protocol number 2019-0115. The studies were conducted in accordance with the local legislation and institutional requirements. The participants provided their written informed consent to participate in this study.
